# Potential effects of valproate and oxcarbazepine on growth velocity and bone metabolism in epileptic children- a medical center experience

**DOI:** 10.1186/s12887-016-0597-7

**Published:** 2016-05-03

**Authors:** Chien-Ming Lin, Hueng-Chuen Fan, Tsu-Yi Chao, Der-Ming Chu, Chi-Chieh Lai, Chih-Chien Wang, Shyi-Jou Chen

**Affiliations:** Department of Pediatrics, Tri-Service General Hospital, National Defense Medical Center, No.325, Cheng-Kung Road, Section 2, Nei-Hu, Taipei 114 Taiwan; Graduate Institute of Medical Sciences, National Defense Medical Center, No.161, Cheng-Kung Road, Section 6, Nei-Hu, Taipei 114 Taiwan; Department of Pediatrics, Tungs’ Taichung MetroHarbor Hospital, No.699, Section 1, Zhongqi Road, Wuqi Township, Taichung, 435 Taiwan; Division of Hematology/Oncology, Department of Internal Medicine, Taipei Medical University, Shuang Ho Hospital, No.291, Zhongzheng Road, Zhonghe District, New Taipei City, 235 Taiwan; Graduate Institute of Clinical Medicine, College of Medicine, Taipei Medical University, No.252, Wu Hsing Street, Taipei, 110 Taiwan

**Keywords:** Antiepileptic drugs, Growth velocity, Bone metabolism, Tartrate-resistant acid phosphatase 5b, Bone-specific alkaline phosphatase

## Abstract

**Background:**

Children with longstanding use of antiepileptic drugs (AEDs) are susceptible to developing low bone mineral density and an increased fracture risk. However, the literature regarding the effects of AEDs on growth in epileptic children is limited. The aim of this study was to investigate the potential effects of valproate (VPA) and/or oxcarbazepine (OXC) therapy on growth velocity and bone metabolism.

**Methods:**

Seventy-three ambulatory children (40 boys and 33 girls) with epilepsy, aged between 1 and 18 years (mean age 9.8 ± 4.1 years), were evaluated for growth velocity before and for 1 year after VPA and/or OXC treatment. The bone resorption marker serum tartrate-resistant acid phosphatase 5b (TRAcP5b) and the bone formation marker serum bone-specific alkaline phosphatase (BAP) were measured post-AEDs therapy for 1 year.

**Results:**

The difference in growth velocity (ΔHt) and body weight change (ΔWt) between pre- and post-AEDs treatment were -1.0 ± 2.8 cm/year (*P* < 0.05) and 0.1 ± 3.9 kg/year (*P* = 0.84), respectively. The study population had serum TRAcP5b-SDS of -1.6 ± 1.2 and BAP-SDS of 1.7 ± 3.7 compared with sex- and age-matched healthy children. Significant correlation between serum TRAcP 5b and BAP activities was noted (*r* = 0.60, *p* < 0.001). There was a positive correlation between growth velocity and serum TRAcP 5b activity after AED treatment (*r* = 0.42, *p <* 0.01). No correlation was found between ΔHt, ΔWt, serum TRAcP 5b, BAP activity and types of AEDs.

**Conclusion:**

Growth velocity was significantly decreased in epileptic children after 1 year of VPA and/or OXC treatment. The effect of VPA and/or OXC therapy on dysregulation of bone metabolism might play a crucial role in physical growth.

**Electronic supplementary material:**

The online version of this article (doi:10.1186/s12887-016-0597-7) contains supplementary material, which is available to authorized users.

## Background

Antiepileptic drugs (AEDs) have been shown to be associated with a lowering of bone mineral density (BMD) in childhood and adolescence [1–3], which are critical periods for bone mineralization [4]. The underlying pathophysiological mechanisms of AEDs on bone metabolism are poorly understood but are probably multifactorial, and include inducing cytochrome p450 enzyme activities [5], increased bone turnover [6], inhibition of osteocalcin [7], and increased urinary loss of calcium and phosphorus.

Along with decreased BMD, the disturbance of physical growth during AEDs treatment is also of great concern to physicians. It has been reported that long-term valproate (VPA) and lamotrigine (LTG) therapy, particularly when combined, is associated with short stature [8]. However, the alteration in growth is postulated to be mediated through immobility, not by the AEDs therapy itself [8]. In addition to the investigations into static height only [8], Rattya J et al. conducted a longitudinal growth analysis to evaluate the effect of VPA, carbamazepine (CBZ), or oxcarbazepine (OXC) therapy on physical growth [9]. Of note, AEDs treatment did not affect linear growth in girls with epilepsy [9]. Taken together, findings regarding the adverse effects of AEDs therapy on growth stature are still inconsistent [8, 9].

Furthermore, the literature regarding the influence of AEDs on dynamic height (growth velocity) in epileptic children is limited, and the effects of AEDs on growth via disregulation of bone mineralization are not fully known. Therefore, we conducted a cross-sectional cohort study to evaluate growth velocity and bone turnover markers in growing Taiwanese children with epilepsy to identify the potential effects of VPA and OXC therapy on decreased growth velocity and disruption of bone metabolism.

## Methods

### Participants and study design

Seventy-three ambulatory outpatients with epilepsy aged 1 to 18 years who visited the Department of Pediatrics of Tri-Service General Hospital in Taiwan were enrolled. Based on the International League Against Epilepsy classification [10], all patients were definitively diagnosed with epilepsy or epileptic syndrome and were taking VPA (*n* = 23), OXC (*n* = 29), or VPA combined with OXC (*n* = 21). All of them were free of seizure for longer than 6 months after AEDs treatment and had an active daily life. Exclusion criteria were acute or chronic disease affecting bone metabolism, significant disabilities such as mental or motor retardation, endocrinologic diseases, taking medications other than AEDs, or any other comorbidity, such as muscular dystrophy, neurological conditions, or recent osteomyelitis. Legal guardians or parents of subjects aged less than 7 years signed an informed consent. Both subjects and their legal guardians or parents had to sign a combination assent/parental permission form written simply and at a comprehensive level appropriate for children between ages 7 to 18. The Ethics Review Committee of Tri-Service General Hospital approved this study.

### Anthropometry

Because growth velocity varies at different stages of life, participants were classified into three subgroups according to pubertal age: pre-puberty (boy 1–8 years, girl 1–7 years), puberty (boy 9–14 years, girl 8–13 years), and post-puberty (boy 15–18 years, girl 14–18 years). Retrospective growth data were gathered only from the records at Tri-Service General Hospital, with an accuracy of 0.5 cm for height and 0.1 kg for weight. Height was measured to the nearest 1.0 mm with the same Harpenden wall-mounted stadiometer and weight to the nearest 0.1 kg on a calibrated weight scale at the clinical examination. The difference in growth velocity and body weight change before and for 1 year after AEDs treatment was expressed as ΔHt and ΔWt, respectively.

### Blood sampling

Upon entry, a blood sample was drawn at 8:00 AM after an overnight fast for the evaluation of plasma aspartate aminotransferase (AST), alanine aminotransferase (ALT), blood urea nitrogen (BUN), creatinine (Cre), alkaline phosphatase (ALP), total calcium (Ca), free Ca, phosphorus (P), and blood hemoglobin (Hgb). Another blood sample was taken for two bone turnover markers, the serum bone resorption marker TRAcP 5b [11, 12] and the bone formation marker BAP activities, after AEDs therapy for 12 months. Serum TRAcP 5b was assessed by a solid phase, immunofixed, enzyme activity assay as described previously [13] with an intra- and inter-assay CV of 5.1 % and 3.9 %, respectively. BAP activity was measured using a commercially available quantitative ELISA kit (Metra BAP EIA kit, Quidel Corp., San Diego, CA) in which serum BAP was immobilized by a specific antibody and its activity measured using 4-nitrophenyl phosphate as the substrate.

### Statistical analyses

Basic data were calculated as mean values with standard deviation. Anthropometry data, serum TRAcP 5b and BAP activities were compared with age- and sex-specific Taiwanese reference values and presented as standard deviation scores (SDS = [*x*-mean]/SD) [14, 15]. The statistical analysis was performed by means of the Student’s *t* test (two-tailed) and one-way analysis of variance (ANOVA) for comparison of continuous variables. Pearson’s correlation analysis was used to estimate the relationships between serum TRAcP 5b, BAP activities, and growth velocity after AEDs treatment. A *p-*value < 0.05 was considered significant.

## Results

Basic demographic and biochemical data of epileptic children at baseline and 1 year post-AEDs treatment are shown in Table 1. The 73 patients (40 boys and 33 girls) had a mean age (± SD) of 9.8 ± 4.1 years, height-SDS of -0.2 ± 1.3, weight-SDS of 0.5 ± 1.8, and body mass index-SDS of 1.1 ± 2.6 [14]. The baseline serum levels of AST/ALT, BUN/Cre, ALP, total Ca, free Ca, P and blood Hgb were all within normal ranges. The mean TRAcP 5b activity was 4.8 ± 1.7 μmol/L/min and the mean BAP activity was 144.4 ± 73.2 μmol/L/min after AEDs treatment for 1 year. Compared with the sex- and age-matched healthy Taiwanese children, TRAcP5b-SDS was -1.6 ± 1.2 and BAP-SDS was 1.7 ± 3.7 [15].

**Table 1:** Demographic and biochemical data of epileptic children at baseline and post-AED treatment^a^

Baseline	
Overall gender (N)	
Male/Female	40/33
Total	73
Age (yrs)	9.8 ± 4.1
Type of AEDs (N)	
Valproate	23
Oxcarbazepine	29
Valproate + Oxcarbazepine	21
Growth evaluation	
Height (cm)	133.5 ± 21.5
Height-SDS	-0.2 ± 1.3
Weight (kg)	35.0 ± 17.0
Weight-SDS	0.5 ± 1.8
Body mass index-SDS	1.1 ± 2.6
Laboratory studies	
Aspartate aminotransferase (U/L)	27.6 ± 7.5
Alanine aminotransferase(U/L)	17.1 ± 7.8
Blood urea nitrogen (mg/dL)	13.0 ± 1.2
Creatinine (mg/dL)	0.5 ± 0.2
Alkaline phosphatase (U/L)	226.8 ± 85.0
Total calcium (mg/dL)	9.5 ± 0.4
Free calcium (mg/dL)	4.9 ± 0.3
Phosphorous (mg/dL)	4.7 ± 0.3
Hemoglobin (g/dL)	13.0 ± 1.2
Post-treatment	
Laboratory studies	
Serum TRAcP5b (umol/L/min)	4.8 ± 1.7
TRAcP5b-SDS	-1.6 ± 1.2
Serum BAP (umol/L/min)	144.4 ± 73.2
BAP-SDS	1.7 ± 3.7
Total calcium (mg/dL)	9.2 ± 0.3
Free calcium (mg/dL)	4.7 ± 0.3

^a^Data presented as mean ± SD

Growth velocity post-AEDs treatment had a decreasing trend in all three subgroups (ΔHt: pre-puberty -1.4 ± 3.3 cm/year; puberty -0.7 ± 2.6 cm/year; post-puberty -0.7 ± 1.8 cm/year) (Table 2). Overall, the difference in growth velocity between pre- and post-AEDs treatment was -1.0 ± 2.8 cm/year (*P <* 0.05). The differences in body weight change between pre- and post-AEDs treatment were 1.2 ± 3.0 kg/year in pre-puberty and -3.3 ± 6.6 kg/year in post-puberty, respectively. Among all participants, there was no significant difference in body weight change between pre- and post-AEDs treatment (*P* = 0.84).

**Table 2:** Growth velocity and body weight change of epileptic children before and after AEDs treatment

Patients	No.		Growth velocity (cm/yr)				Body weight change (kg/yr)			
	M/F	Total	Pre-AEDs	Post-AEDs	ΔHt^a^	*p*-value*	Pre-AEDs	Post-AEDs	ΔWt^b^	*p*-value*
Pre-puberty	19/9	28	6.7 ± 3.4	5.3 ± 1.3	-1.4 ± 3.3	0.13	1.9 ± 1.8	2.7 ± 2.0	1.2 ± 3.0	0.33
Puberty	13/17	30	6.5 ± 3.4	5.8 ± 2.0	-0.7 ± 2.6	0.30	3.9 ± 2.8	4.7 ± 2.7	0.5 ± 3.0	0.29
Post-puberty	8/7	15	3.7 ± 2.8	3.0 ± 1.9	-0.7 ± 1.8	0.35	4.3 ± 7.4	1.0 ± 2.3	-3.3 ± 6.6	0.27
Overall	40/33	73	6.1 ± 3.4	5.2 ± 2.0	-1.0 ± 2.8	0.04	3.2 ± 3.6	3.4 ± 2.7	0.1 ± 3.9	0.84

Data presented as mean ± SD; ^a^The difference in growth velocity between pre- and post-AEDs treatment; ^b^The difference in body weight change between pre- and post-AEDs treatment; *P-value between pre- and post-AEDs treatment

There was a significant correlation between serum TRAcP 5b and BAP activity after AED treatment (*r* = 0.60, *p* < 0.001) (data not shown). In Table 3, a positive correlation between growth velocity after AEDs treatment and serum TRAcP 5b activity was noted in the post-pubertal group (*r* = 0.93, *p* < 0.01) and in all patients (*r* = 0.42, *p <* 0.01). No correlation was found between ΔHt, ΔWt, serum TRAcP 5b, BAP activity and types of AEDs (data not shown).

**Table 3:** Correlation of coefficient between growth velocity after AEDs treatment and bone turnover markers

	Growth velocity after AEDs treatment			
	Pre-puberty	Puberty	Post-puberty	Overall
Serum TRAcP5b	0.07	0.23	0.93*	0.42*
Serum BAP	0.04	0.01	0.49	0.22

*P < 0.01

## Discussion

Our findings of a decrease in the bone resorption marker TRAcP 5b-SDS (-1.6 ± 1.2), an increase in the bone formation marker BAP-SDS (1.7 ± 3.7), and a significant correlation between serum TRAcP 5b and BAP activities (*r* = 0.60, *p* < 0.001) might be indirect evidence of increased bone turnover. In terms of body height, a decreased growth velocity after VPA and/or OXC therapy for 1 year (-1.0 ± 2.8 cm/year, *p* < 0.05) and a significantly positive correlation between growth velocity and serum TRAcP 5b activity after AEDs therapy (*r* = 0.42, *p* < 0.01) were observed in all subjects. These interesting findings might partly explain the crucial role of the uncoupling of bone metabolism in decelerated physical growth in growing children with short-term VPA and/or OXC treatment. To our knowledge, the present study is the first report to delineate the relationship between decreased growth velocity and the short-term use of VPA and/or OXC therapy in a representative Taiwan population-based sample. However, a well-controlled longitudinal study with more patients and evaluation of more bone turnover markers is needed to confirm our hypothesis.

Both enzyme-inducing and non-enzyme inducing AEDs can cause abnormalities in bone metabolism [16–19]. The negative influence of AEDs on BMD is complex and not understood completely, but several lines of evidence suggest a role for the induction of cytochrome p450 enzyme activities, which accelerate vitamin D hydroxylation to inactive forms [5], increased bone turnover [6], inhibition of osteocalcin [7], and decreased intestinal transport of calcium [20]. Besides decreased BMD, the influence of AEDs treatment on physical growth is of great concern in clinical practice nowadays. However, the literature regarding the adverse effect of AEDs therapy on growth stature is very limited and the results are inconsistent [8, 9]. Guo et al. reported long-term VPA and LTG therapy, particularly when combined, is associated with short stature, low BMD, and reduced bone formation; but these alterations are thought to be mediated primarily through reduced physical activity rather than though a direct link to AEDs therapy [8]. In contrast to the investigation of static height alone [8], our study found epileptic children with normal daily activity had decreased dynamic growth patterns associated with increased bone turnover after short-term VPA and/or OXC treatment. Although Rattya et al. reported VPA, CBZ, or OXC seem not to have an adverse effect on linear growth in girls with epilepsy, VPA-related weight gain could be observed in that series [9]. As illustrated, weight gain induced advanced bone age resulting in rapid growth may partially mask the true effect of VAP on growth in that series. On the contrary, our three study subgroups all had a trend toward a decrease in growth velocity, no matter whether body weight increased or not. There was a lack of obvious weight gain in our study, which is typically associated with a protective effect on bone mineralization [21], so we supposed that the decelerated growth velocity might not be attributed to body weight change, but to AEDs treatment itself. Nevertheless, the effect of the interaction between AEDs therapy and genetic, hormonal, and environmental factors on physical growth in Taiwanese epileptic children still should be considered.

In adults and children, VPA therapy is associated with weight gain, high body fat, and hyperleptinaemia [9, 22]. Thus, it is speculated that VPA could affect bone mass accrual and/or turnover indirectly through weight gain and the associated hyperleptinaemia [19]. However, the AEDs-induced weight gain was only observed in patients taking VPA, not in those treated with OXC [9]. In contrast to previous reports [9, 22], the differences in body weight change between pre- and post-VPA and/or OXC treatment did not alter significantly in the present study (*p* = 0.84). Furthermore, our study found no correlation between ΔWt and usage of types of AEDs (data not shown). We supposed that the effect of VPA on weight gain in all participants might be masked by decreased body weight gain in the post-pubertal subgroup, in consideration of the elevated self-image and sense of self-esteem, as well as the lack of body weight change with OXC therapy [9]. Of importance, the weight gain in patients treated with VPA was slow but progressive [9], which may subsequently increase weight-bearing for bone remodeling, BMD, and linear growth. Therefore, the possibility of weight gain in epileptic patients should be monitored during long-term VPA treatment to elucidate its true effect on bone growth.

Serum vitamin D levels were not measured in our study. Vitamin D deficiency in participants with epilepsy was reported to be common in some, but not all, earlier studies [19, 23–25]. These discrepant results may be attributed to whether AEDs, with their cytochrome P450 enzyme inducers, which increase vitamin D metabolism, were used or not. On the other hand, confounding factors such as AEDs polytherapy and immobility may also contribute to vitamin D deficiency/insufficiency in epileptic patients [8, 26]. In contrast to strong enzyme inducers such as CBZ, non-enzyme-inducing (VPA) and minimal enzyme-inducing (OXC) agents were used by our participants. All subjects in the present study were ambulatory and normally physically active. Furthermore, Taiwan is a subtropical Asian country and so sunlight exposure was also adequate. Herein, disruption to bone metabolism related to vitamin D deficiency seemed not to be an important contributor to decreased growth velocity in our series.

Nevertheless, our study has some limitations. Firstly, there was a lack of X-ray image to investigate the relationship between BMD, bone age study and growth velocity. However, due to the fear of high-level exposure to radiation, the risk-benefit analysis did not justify performing BMD measurements in present study, particularly from a parental point of view. Furthermore, findings regarding the effect of AEDs on BMD are still conflicting [23, 24, 26–28]. Secondly, the functions of insulin like growth factor-I (IGF-I) were potentially to increase markers of osteoblastic activity and reduce bone resorption [29]; and serum IGF-binding proteins-2 (IGFBP-2) levels was associated with low BMD and high bone resorption markers [30, 31]. However, the growth hormone (GH)/IGF axis, which is essential to maintain homeostasis of bone turnover, was not evaluated in our study [32]. Finally, our patient sample size was small and was not equally distributed across age subgroups, which might explain why the differences in growth velocity between pre- and post-AEDs treatment were significant overall, but not in each subgroup. Therefore, a larger-scale prospective study in consideration of IGF-I, IGFBP-2, bone turnover biomarkers, and growth velocity is necessary to clarify our hypothesis of AEDs on growth and bone metabolism.

## Conclusion

Our findings suggest that 1-year survey of VPA and/or OXC therapy among growing epileptic children might be associated with decreased growth velocity. This alteration seems to be mediated through the effect of AEDs on dysregulation of bone metabolism. To monitor growth velocity and bone metabolism, a larger-scale, long-term prospective study considering weight gain effect of AEDs, genetic, hormonal, and nutritional factors is needed to elucidate the true effects of AEDs itself on physical growth.

## Availability of data and materials

The datasets supporting the conclusions of this article is included within the manuscript and additional file 1.

## Additional file

Additional file 1: The orginal data of the study. (XLSX 19 kb)

## Abbreviations

AEDs: antiepileptic drugs
BAP: bone-specific alkaline phosphatase
BMD: bone mineral density
CBZ: carbamazepine
LTG: lamotrigine
OXC: oxcarbazepine
TRAcP5b: tartrate-resistant acid phosphatase 5b
VPA: valproate
